# Microbial Analysis of Umbilical Cord Blood Reveals Novel Pathogens Associated with Stillbirth and Early Preterm Birth

**DOI:** 10.1128/mbio.02036-22

**Published:** 2022-08-22

**Authors:** Emilie L. Vander Haar, Guojun Wu, Cynthia Gyamfi-Bannerman, Charlene Thomas, Ronald J. Wapner, Uma M. Reddy, Liping Zhao, Robert M. Silver, Robert L. Goldenberg, Yiping W. Han

**Affiliations:** a Department of Obstetrics and Gynecology, Vagelos College of Physicians and Surgeons, Columbia University grid.239585.0grid.21729.3fgrid.239585.0 Irving Medical Center, New York, New York, USA; b Division of Maternal Fetal Medicine, Department of Obstetrics and Gynecology, Weill Cornell Medicine, New York, New York, USA; c Department of Biochemistry and Microbiology, School of Environmental and Biological Sciences and Center for Microbiome, Nutrition, and Health, New Jersey Institute for Food, Nutrition, and Health, Rutgers, The State University of New Jersey, New Brunswick, New Jersey, USA; d Department of Obstetrics, Gynecology, and Reproductive Sciences, UC San Diego, La Jolla, California, USA; e Division of Biostatistics, Department of Population Health Sciences, Weill Cornell Medicine, New York, New York, USA; f Department of Obstetrics and Gynecology, University of Utah, Salt Lake City, Utah, USA; g Section of Oral, Diagnostics and Rehabilitation Sciences, College of Dental Medicine, Columbia University grid.239585.0grid.21729.3fgrid.239585.0 Irving Medical Center, New York, New York, USA; h Department of Microbiology and Immunology, Vagelos College of Physicians and Surgeons, Columbia University grid.239585.0grid.21729.3fgrid.239585.0 Irving Medical Center, New York, New York, USA; i Institute of Human Nutrition, Columbia University grid.239585.0grid.21729.3fgrid.239585.0 Irving Medical Center, New York, New York, USA; Iowa State University

**Keywords:** stillbirth, preterm birth, cord blood, microbiome, 16S rRNA, amplicon sequence variants, oral bacteria, *Streptococcus agalacticae*, *Fusobacterium*, PCR, group B streptococcus, human microbiome

## Abstract

Stillbirths account for half of all perinatal mortality, but the underlying cause of a significant portion of the cases remains unknown. We set out to test the potential role and extent of microbial infection in stillbirth through a case-control analysis of fetal cord blood collected from the multisite Stillbirth Collaborative Research Network. Cases (*n* = 60) were defined as stillbirths at >20 weeks of gestation, and controls (*n* = 176) were live births. The bacterial presence, abundance, and composition were analyzed by endpoint PCR of full-length 16S rRNA and the V4 amplicon sequence variants (ASVs). The results demonstrate that bacterial prevalence and abundance were both significantly increased in stillbirth, even after adjusting for maternal age, race, body mass index, number of pregnancies, gestational age, and multiple gestations. Composition of bacterial communities in the cord blood also differed significantly. Using a group of 25 ASVs differentially abundant between the two groups, a Random Forest classification model achieved an accuracy score of 0.76 differentiating stillbirth and live birth, with Group B *Streptococcus* as the most enriched species in stillbirth. Positive PCR was also significantly associated with early preterm birth. A group of oral anaerobes, including *Actinomyces*, Campylobacter*, Fusobacterium, Peptostreptococcus, Porphyromonas*, and *Prevotella,* were enriched in live early preterm birth, suggesting possible oral origin of infection. Our ASV-based microbiome analysis revealed specific candidate pathogens associated with infections in stillbirth and early preterm birth. The cord blood microbial signatures may be markers of adverse pregnancy outcomes. Our study will help identify possible mechanism of infection and improve our ability to prevent stillbirth and early preterm birth.

## INTRODUCTION

Stillbirth accounts for half of all perinatal mortality. In the U.S. there are approximately 21,000 stillbirths per year, at a rate of 5.7 fetal deaths per 1,000 births (1). The cause for a significant portion of all stillbirths remains unknown (2, 3). Although maternal and fetal infections are believed to account for some cases, the true prevalence of microbial infection in stillbirth is unclear (4, 5). Currently, diagnosis of infection requires histologic examination of placenta, microbial culture, and fetal autopsy, which are not always performed (6). Even when fetal or placental cultures are performed, important infections may be missed, as not all microorganisms are easily identified by routine culturing methods. This is especially true for certain microorganisms, e.g., Fusobacterium nucleatum, a Gram-negative anaerobe that is difficult to detect by routine culture yet known to be associated with adverse pregnancy outcomes such as preterm birth, stillbirth, and early-onset neonatal sepsis (7–10). Polymerase chain reaction (PCR) based culture-independent microbial detection methods have been validated in multiple studies and have been used to identify clinically relevant uncultivated and difficult-to-cultivate microbial species (7, 8, 10).

While the vaginal and placental microbiomes have been extensively studied, the cord blood microbiome has been mostly untapped. A previous study reported anecdotal detection of microbial species in umbilical cord blood in cases of preterm birth and early-onset neonatal sepsis (10), suggesting that this easily attainable biospecimen may be useful for detection of microbial infection associated with adverse pregnancy outcomes. Here, we report an extensive case-control analysis of umbilical cord blood samples using both endpoint PCR amplification of the full-length 16S rRNA gene and Illumina sequencing of the V4 region. We show that bacterial DNA presence and signature within cord blood are associated with stillbirth and early preterm birth and vary by gestational age.

## RESULTS

### Demographic and clinical characteristics of live births and stillbirths.

There was no significant difference in baseline demographics between cases and controls, including maternal age, race, parity, body mass index, or education level. There were also no significant differences in the rates of diabetes, smoking, chronic hypertension, maternal illicit drug use, mode of delivery, presence of preterm premature rupture of membranes (PPROM), antibiotics given prior to delivery, or fetal sex. The live birth group had higher rates of preterm labor and spontaneous labor than the stillbirth group. The stillborn infants were more likely to be born at an earlier gestational age, weighed less at birth, and had higher rates of meconium in the amniotic fluid (Table 1).

**TABLE 1:** Patient characteristics^*a*^

Characteristics	Stillbirth (*n* = 60)	Liveborn (*n* = 176)	*P* value
Demographic characteristics			
Maternal age (yrs; mean ± SD)	28 ± 6	27 ± 6	0.11
Race			0.92
Caucasian	24 (40%)	75 (43%)	
African American	4 (7%)	13 (7%)	
Hispanic	26 (43%)	75 (43%)	
Other	6 (10%)	13 (7%)	
Multiparity	35 (58%)	119 (68%)	0.19
BMI			0.22
<18.5	0 (0%)	6 (100%)	
18.5–24.9	27 (46%)	92 (54%)	
25–29.9	17 (29%)	39 (23%)	
30–34.9	10 (17%)	16 (9%)	
>35	5 (9%)	19 (11%)	
Education Level			0.49
0–11 yrs	14 (25%)	49 (29%)	
12 yrs	11 (19%)	41 (24%)	
13+ yrs	32 (56%)	80 (47%)	
Multiple gestation	2 (3%)	16 (9%)	0.26
Illicit drug use	19 (33%)	47 (28%)	0.46
Diabetes	3 (5%)	6 (3%)	0.70
Smoking	8 (14%)	18 (11%)	0.48
Chronic hypertension	5 (8%)	11 (6%)	0.56
Preeclampsia	3 (6%)	25 (15%)	0.08
Obstetric characteristics			
Antibiotics prior to delivery	17 (65%)	69 (74%)	0.38
PPROM	9 (23%)	18 (19%)	0.61
Preterm labor	8 (21%)	42 (45%)	**0.01**
Spontaneous labor	18 (32%)	95 (54%)	**0.001**
Vaginal delivery	45 (75%)	121 (70%)	0.42
Meconium	15 (29%)	12 (8%)	**<0.0001**
Clinical chorioamnionitis	5 (14%)	5 (5%)	0.14
Histologic chorioamnionitis	14 (27%)	23 (15%)	0.06
Birthweight (grams; mean ± SD)	2076 ± 1279	2715 ± 1032	**<0.0001**
Male sex	32 (53%)	101 (57%)	0.27
Gestational age (wks; mean ± SD)	32 ± 7	35 ± 5	**0.0006**

a Data are presented as *n* (percentage) unless otherwise indicated. PPROM, preterm premature rupture of membranes. *P* values <0.05 are boldfaced.

Both clinical and histologic chorioamnionitis were noted to be more prevalent in stillbirths, but the differences did not reach significance (Table 1). After adjusting for potential confounding factors, including maternal age, race, parity, BMI, gestational age, and multiple gestation, neither clinical chorioamnionitis nor histologic chorioamnionitis was significantly different between the two groups (Table 2).

**TABLE 2:** Association of measures of infection with stillbirth and early preterm birth^*a*^

Birth outcome groups	Clinical chorioamnionitis	Histologic chorioamnionitis	PCR+	TRC
Stillbirth OR [95% CI]	2.8 [0.7–10.7]	2.0 [0.9–4.2]	7.0 [3.6–13.9]	1.5 [1.3–1.8]
*P* value	0.12	0.076	**<0.001**	**<0.001**
Stillbirth aOR [95% CI]	3.2 [0.7–14.8]^*b*^	1.3 [0.5–3.3]^*c*^	7.0 [3.3–15.2]^*b*^	1.6 [1.3–2.0]^*b*^
*P* value	0.13	0.58	**<0.001**	**<0.001**
Early PTB OR [95% CI]	4.7 [1.2–23.0]	10.3 [4.6–23.8]	2.6 [1.3–5.2]	1.3 [1.1–1.5]
*P* value	**0.03**	**<0.001**	**0.005**	**0.002**
Early PTB aOR [95% CI]	3.8 [1.0–18.7]^*d*^	9.5 [4.2–22.5]^*d*^	2.5 [1.2–5.1]^*e*^	1.3 [1.1–1.5]^*d*^
*P* value	0.067	**<0.001**	**0.01**	**0.006**

a OR, odds ratio; CI, confidence interval; aOR, adjusted odds ratio; early PTB, preterm birth <32 weeks of gestation (live birth and stillbirth); TRC, total ASV read counts (log-transformed). *P* values <0.05 are boldfaced.

b aOR adjusted for maternal age, race, BMI, parity, multifetal gestation, and gestational age.

c aOR adjusted for maternal age, race, BMI, parity, and gestational age.

d aOR adjusted for maternal age and parity.

e aOR adjusted for maternal age, race, parity, and multifetal gestation.

### Bacterial detection rate and abundance in umbilical cord blood are significantly higher in stillbirths and in early preterm birth.

End-point PCR amplification of the full-length 16S rRNA gene was performed to examine the presence of bacterial DNA. Detection of the 16S rRNA gene (hereafter, “PCR+”) was significantly higher in stillbirths than live births (Table 2). After adjusting for maternal age, race, parity, BMI, gestational age, and multiple gestation, the PCR+ rate was still significantly higher in stillbirths than live births. These results suggest that PCR analysis of bacterial DNA in umbilical cord blood may be used to detect possible fetal infections in cases of stillbirth, as opposed to histopathological or clinical diagnoses of intrauterine infection, which showed no difference between stillbirth and live birth (Table 2).

When the subjects were further divided according to gestational age into early preterm birth (<32 weeks of gestation), preterm birth (32–36 weeks), and term birth (>37 weeks), the PCR+ rate was significantly higher among stillbirths than live births in each gestational age group (Table 3). Furthermore, the PCR+ results were significantly associated with early preterm births of <32 weeks of gestation when stillbirths and live births were combined. After adjusting for available confounding factors including maternal age, race, parity, multiple gestation, and/or gestational age, PCR positivity remained significantly associated with early preterm birth (Table 2).

**TABLE 3:** Presence and quantity of bacterial DNA are associated with stillbirth, even when stratified by gestational age

Birth outcome groups	Stillbirths (*n* = 60)	Live births (*n* = 176)	*P* value^*a*^
Positive rate of end-point PCR of full-length 16S rRNA^*b*^			
<32 wks	12 (60%)	7 (22%)	**0.013**
32–36 wks	8 (50%)	5 (12%)	**0.003**
≥37 wks	10 (42%)	10 (10%)	**0.001**
Median total 16S read counts (log-transformed) of V4 region^*c*^			
<32 wks	9.83 [7.30, 10.97]	6.90 [5.72, 8.61]	**0.016**
32–36 wks	7.87 [6.63, 8.39]	6.33 [5.42, 7.32]	**0.005**
≥37 wks	7.71 [6.43, 10.16]	6.31 [5.65, 7.33]	**0.001**

a *P* values <0.05 are boldfaced.

b Data presented as *n* (%).

c Data presented as median total read count [interquartile range].

To quantify the bacterial abundance and to characterize the microbial community in the umbilical cord blood, the V4 regions of 16S rRNA were sequenced on the Illumina MiSeq platform. In addition to the cord blood samples of stillbirths (cases) and live births (controls), we also included 12 blank buffer controls. A total of 4,651 amplicon sequence variants (ASVs) were identified after denoising by DADA2 (11). To remove ASVs artificially generated during PCR and sequencing, we applied a bootstrapping-based abundance filtering method (12) and removed 424 spurious ASVs with no more than 5 reads across all samples. For samples containing an overwhelming ratio of human to bacterial DNA, off-target amplification of human DNA by 16S rRNA gene primers has been reported (13). Thus, we aligned the 4,227 reliable ASVs against human genome and nr database using NCBI BLASTN to identify and remove 1,253 nonmicrobiota ASVs. Moreover, 76 ASVs of potential buffer contaminants were identified and removed using the decontam R package (14).

The total ASV read counts were significantly higher in stillbirths compared to live births (Table 2). The difference remained significant after adjusting for maternal age, race, BMI, parity, gestational age, and multiple gestations. Consistent with PCR results, the total read counts were significantly higher among stillbirths than live births in all three gestational groups (<32 weeks, 32–36 weeks, and ≥37 weeks; Table 3). Higher total read counts were also significantly associated with all early preterm births (live birth + stillbirth). After adjusting for possible confounders, the relationship remained significant (Table 2). These results indicate that the bacterial abundance in umbilical cord blood, and not just presence, is associated with stillbirth and varied by gestational age.

### Microbial signature in umbilical cord blood is associated with stillbirth.

To compare the microbial composition between samples, we downsized each sample to 1,000 reads, which corresponded to 1,875 reliable microbial ASVs from 39 cases (stillbirth) and 66 control (live birth) samples in the subsequent analysis.

**FIG 1:**
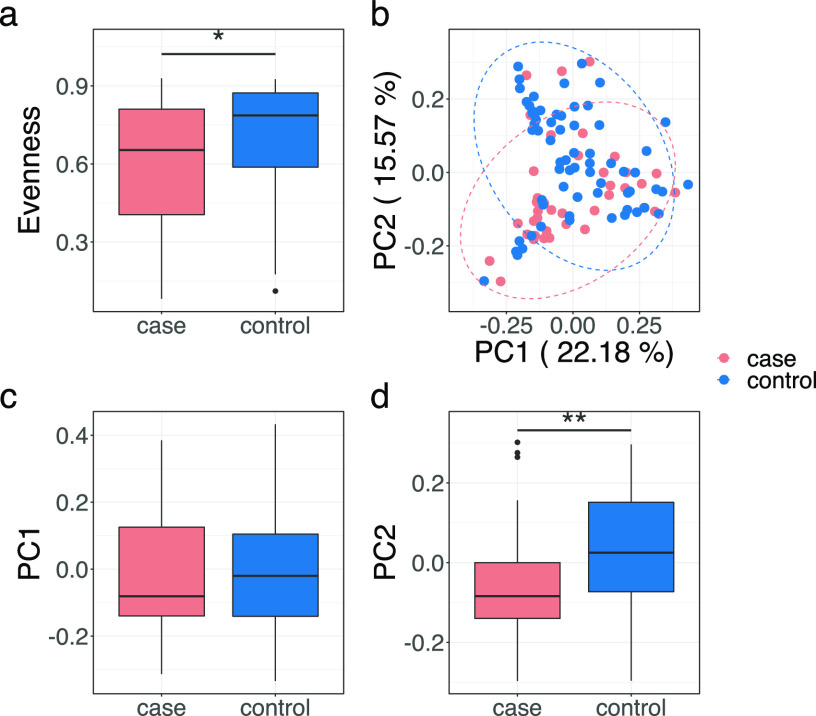
Different microbial composition of umbilical cord blood between stillbirth (case) and live birth (control). (a) Live birth had higher evenness than stillbirth. (b) Principal coordinates analysis (PCoA) plot based on weighted UnFirac distance. (c) PC1 of PCoA plot. (d) PC2 of PCoA plot. Mann-Whitney test (two-sided) was applied to compare cases (*n* = 39) and controls (*n* = 66). *, *P* < 0.05; **, *P* < 0.01. Boxes show the medians and the interquartile ranges (IQRs), the whiskers denote the lowest and highest values that were within 1.5 times the IQR from the first and third quartiles, and outliers are shown as individual points.

**FIG 2:**
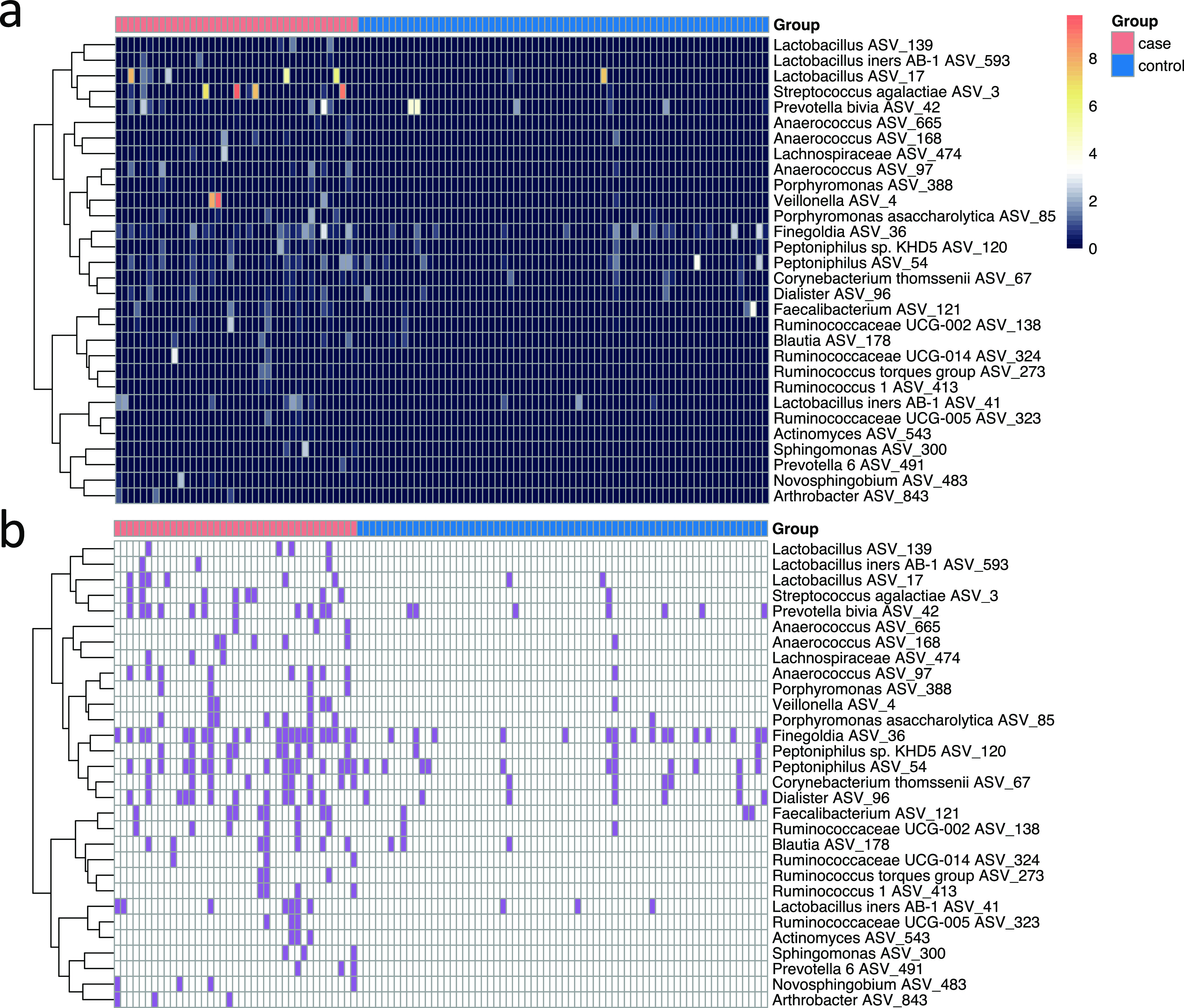
Thirty differential ASVs between stillbirth (case) and live birth (control). (a) Heatmap of the relative abundance (square root transformed) of the 30 ASVs, which had significantly different relative abundance (Mann-Whitney test [two-sided], *P* < 0.05) and prevalence (Fisher’s exact test [two-sided], *P* < 0.05) between the case and control groups. The clustering of the ASVs is based on the Spearman correlations of the abundance and ward linkage. (b) Binary heatmap shows the presence (purple color) and absence (white color) of the 30 AVSs across case (*n* = 39) and control (*n* = 66) samples.

**FIG 3:**
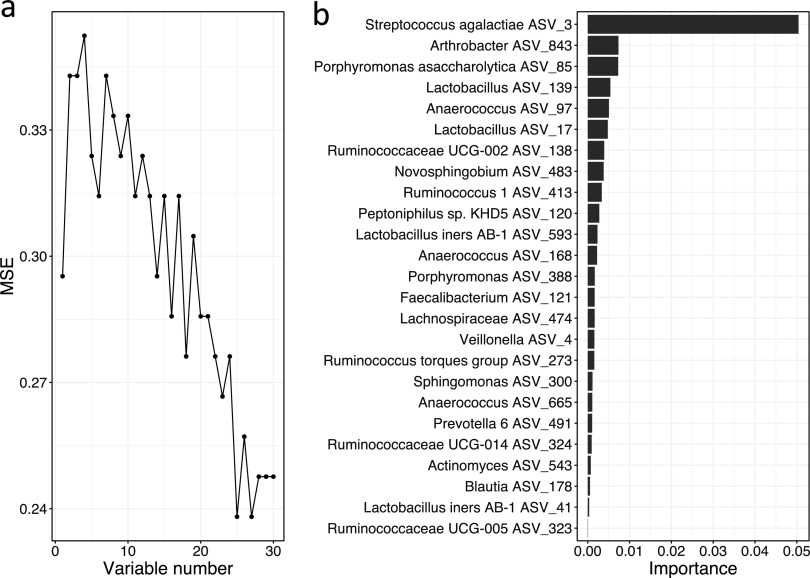
Microbiota-based model to predict stillbirth (case) and live birth (control). Classification model based on Random Forest algorithm by using the abundance of the 30 ASVs. (a) Number of variables and mean squared error of the corresponding model. (b) The Random Forest model assigns a mean error rate or feature-importance score to each feature; this value indicates the extent to which each predictor contributes to the accuracy of the model. This model achieves an accuracy score of 0.76.

Compared to the live birth group, the microbiota in umbilical cord blood samples from the stillbirth group had significantly lower diversity, as evidenced by a reduced evenness (*P* = 0.039) (Fig. 1a). There was no difference in Shannon index and ASV numbers between the two groups (Fig. S1). Principal coordinates analysis (PCoA) based on the weighted UniFrac distance showed a significant difference in the overall umbilical cord microbiota structure between the case and control groups along PC2 (Fig. 1b–d, *P* = 0.0017), and a PERMANOVA test also showed a significant difference (*P* = 0.007, R^2^ = 0.025). A significant but smaller difference between the case and control groups was observed based on unweighted UniFrac distance as well (Fig. S2), as evidenced by a significant difference along PC1 in the PCoA plot (*P* = 0.021) and a significant PERMANOVA test result (*P* = 0.036, R^2^ = 0.014). At the ASV level, we found 30 ASVs with both different relative abundance (Mann-Whitney test) and prevalence (Fisher’s exact test) between the case and control groups (Fig. 2). While all 30 ASVs showed higher prevalence in the stillbirth group, 14 ASVs were detected exclusively in stillbirth, absent in live birth (Table 4). To explore if the 30 ASVs can be used as biomarkers to differentiate stillbirth and live births, we applied a Random Forest Classification model using the 30 ASVs as predicters. Based on a 10-fold cross-validation and feature selection process, the best classification model was obtained, with a maximum accuracy score of 0.76 based on 25 ASVs (Fig. 3). Notably, Streptococcus agalactiae ASV_3, detected in 20.51% cases and 1.52% controls, contributed most to the model, and its importance was much greater than the remaining 24 ASVs (Table 4). S. agalactiae, also known as group B Streptococcus (GBS), has been reported as a high-risk factor in stillbirth (15). The second contributor, *Arthrobacter* ASV_843, was detected in 7.69% cases and undetected in the controls. *Arthrobacter* has been reported in a case of intrauterine fetal death (16). Eight additional ASVs belong to taxa in which members have been previously detected in reproductive and other infections (Table 4). These findings support the validity of our model.

**TABLE 4:** Detection of 25 ASVs used in the best prediction model in stillbirths and live births^*a*^

ASV (in order of importance)	Detection in cases (%)	Detection in controls (%)	Previous implication in reproductive infections	References
Streptococcus agalactiae ASV_3	20.51	1.52	Stillbirth	Sealed et al. (5)
*Arthrobacter* ASV_843	7.69	—	Intrauterine fetal death	Shigeta et al. (6)
Porphyromonas asaccharolytica ASV_85	17.95	1.52	Associated with infection	Acuna-Amador et al. (7)
*Lactobacillus* ASV_139	10.26	—		
*Anaerococcus* ASV_97	20.51	1.52	Bacterial vaginosis	Africa et al. (8)
*Lactobacillus* ASV_17	15.38	3.03		
*Ruminococcaceae* UCG-002 ASV_138	15.38	3.03		
*Novosphingobium* ASV_483	10.26	—	In placenta of newborns with fetal macrosomia	Zheng et al. (9),Salihu et al. (10)
*Ruminococcus* 1 ASV_413	10.26	—		
*Peptoniphilus* sp. KHD5 ASV_120	25.64	4.55	Bacterial vaginosis	Africa et al. (8)
Lactobacillus iners AB-1 ASV_593	7.69	—		
*Anaerococcus* ASV_168	12.82	1.52	Bacterial vaginosis	Africa et al. (8)
*Porphyromonas* ASV_388	10.26	—	Associated with infection	Acuna-Amador et al. (7)
*Faecalibacterium* ASV_121	20.51	4.55		
*Lachnospiraceae* ASV_474	7.69	—		
*Veillonella* ASV_4	12.82	1.52		
Ruminococcus torques *group* ASV_273	7.69	—		
*Sphingomonas* ASV_300	7.69	—	Stillbirth	Madhi et al. (28)
*Anaerococcus* ASV_665	7.69	—	Bacterial vaginosis	Africa et al. (8)
*Prevotella* 6 ASV_491	7.69	—		
*Ruminococcaceae* UCG-014 ASV_324	7.69	—		
*Actinomyces* ASV_543	7.69	—		
*Blautia* ASV_178	17.95	4.55		
Lactobacillus iners AB-1 ASV_41	17.95	4.55		
*Ruminococcaceae* UCG-005 ASV_323	7.69	—		

*a*—, not detected. The ASVs were sorted in descending order of importance in the Random Forest model.

10.1128/mbio.02036-22.1 FIG S1 Comparison of (a) Shannon index and (b) ASV number between stillbirth (case) and live birth (control). Mann-Whitney test (two-sided) was applied to compare cases (*n* = 39) and controls (*n* = 66). Boxes show the medians and the interquartile ranges (IQRs), the whiskers denote the lowest and highest values that were within 1.5 times the IQR from the first and third quartiles, and outliers are shown as individual points. Download FIG S1, PDF file, 0.03 MB. Copyright © 2022 Vander Haar et al. 2022 Vander Haar et al. https://creativecommons.org/licenses/by/4.0/ This content is distributed under the terms of the Creative Commons Attribution 4.0 International license.

10.1128/mbio.02036-22.2 FIG S2 Different microbial composition of umbilical cord blood between stillbirth (case) and live birth (control) based on unweighted UniFrac distance. (a) Principal coordinates analysis (PCoA) plot based on unweighted UnFirac distance. (b) PC1 of PCoA plot. (c) PC2 of PCoA plot. Mann-Whitney test (two-sided) was applied to compare case (*n* = 39) and control (*n* = 66). *, *P* < 0.05; **, *P* < 0.01. Boxes show the medians and the interquartile ranges (IQRs), and the whiskers denote the lowest and highest values that were within 1.5 times the IQR from the first and third quartiles. Download FIG S2, PDF file, 0.03 MB. Copyright © 2022 Vander Haar et al. 2022 Vander Haar et al. https://creativecommons.org/licenses/by/4.0/ This content is distributed under the terms of the Creative Commons Attribution 4.0 International license.

### Microbiome signature in umbilical cord blood varied by gestational age.

**FIG 4:**
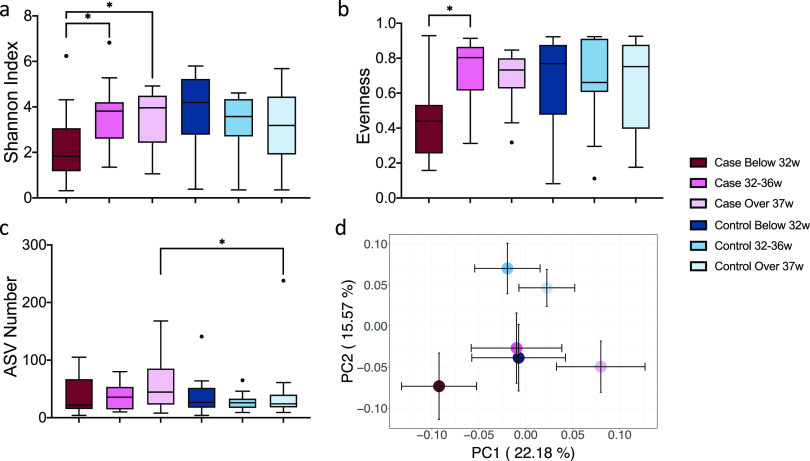
Umbilical cord blood microbiome varies by gestational age. (a) Shannon Index. (b) Evenness Index. (c) ASV number. (d) Principal coordinates analysis (PCoA) plot based on weighted UnFirac distance. Each point in the PCoA plot represents the mean (± SEM) principal coordinate (PC) score of all individual subjects. (a–c) Boxes show the medians and the interquartile ranges (IQRs), the whiskers denote the lowest and highest values that were within 1.5 times the IQR from the first and third quartiles, and outliers are shown as individual points. Mann-Whitney test (two-sided) was applied to compare the case and control groups at different gestational age stage and compare different gestational age stages within the case and control groups. Cases (stillbirth): <32 weeks *n* = 15, 32–36 weeks *n* = 10, ≥37 weeks *n* = 14; controls (live birth): <32 weeks *n* = 16, 32–36 weeks *n* = 15, ≥37 weeks *n* = 35.

The case and control samples were stratified based on gestational age. In the stillbirth group, significantly decreased microbial diversity was observed in the early preterm group (<32 weeks), with both a lower Shannon index (*P* = 0.036 compared to 32–36 weeks, *P* = 0.023 compared to ≥37 weeks) (Fig. 4a) and a lower evenness (*P* = 0.026 compared to 32–36 weeks) (Fig. 4b). In the live birth group, there was no difference in microbial diversity between the three gestational age groups, measured by either Shannon index or evenness (Fig. 4a and b). Among early preterm and preterm groups, there was no difference in alpha diversity between stillbirth and live birth. However, for term births, the stillbirth group had a significantly higher ASV number than the live birth group (*P* = 0.033) (Fig. 4c). These results demonstrate that umbilical cord blood microbiome diversity varies by gestational age in stillbirth, but not in live birth.

Analysis of the microbiome composition using PERMANOVA tests based on weighted UniFrac distance showed that gestational age was significantly correlated with the overall microbial structure across all samples (*P* = 0.011, R^2^ = 0.035). PCoA plot showed that the microbial structure in the stillbirth group changed along PC1 (<32 weeks versus ≥37 weeks: *P* = 0.010), while in live birth it changed along PC2 (<32 weeks versus 32–36 weeks: *P* = 0.037; <32 weeks versus ≥37 weeks: *P* = 0.063) (Fig. 4d). Significant separations between the stillbirth and live birth groups were observed along PC2 for 32–36 weeks (*P* = 0.048) and ≥37 weeks (*P* = 0.021) (Fig. 4d). Therefore, the umbilical cord blood microbiome composition differs not only between stillbirth and live birth, but also by gestational age.

Differences between stillbirth and live birth were detected at the ASV level at different gestational stages (Table 5). Among the early preterm births (<32 weeks), three ASVs showed significantly higher prevalence in the stillbirth group than the live birth group. The Random Forest Classification model based on 2 out of the 3 ASVs had an accuracy score of 0.74 to distinguish between the two groups (Fig. S3). For gestational age of 32–36 weeks, one differential ASV was identified. For the group at ≥37 weeks, 24 differential ASVs were detected. The Random Forest Classification model based on 19 out of 24 ASVs reached an accuracy score of 0.78 to distinguish between case and control (Fig. S4).

**TABLE 5:** ASVs in umbilical cord blood differentially detected between cases (stillbirth) and controls (live birth) in different gestational age groups

ASV^*a*^	Stillbirth (%)^*b*^	Live birth (%)^*b*^	*P* value^*c*^
<32 wks			
*Burkholderiaceae* ASV_14	40.00	6.25	0.04
*Cloacibacterium* ASV_35	26.67	—	0.04
Lactobacillus iners AB-1 ASV_41	26.67	—	0.04
32–36 wks			
*Peptoniphilus* sp. KHD5 ASV_120	40.00	—	0.02
>37 wks			
*Anaerococcus* ASV_665	21.43	—	0.02
*Anaerococcus* ASV_88	35.71	5.71	0.02
*Anaerococcus* ASV_97	28.57	—	—
*Bacteroides* ASV_221	28.57	5.71	0.05
*Bacteroides* ASV_46	35.71	8.57	0.03
*Blautia* ASV_163	28.57	5.71	0.05
*Blautia* ASV_178	50	5.71	—
Campylobacter ureolyticus ASV_79	28.57	2.86	0.02
*Dialister* ASV_96	42.86	8.57	0.01
*Dorea* ASV_118	35.71	8.57	0.03
*Faecalibacterium* ASV_121	42.86	5.71	—
*Fastidiosipila* ASV_403	21.43	—	0.02
*Fenollaria timonensis* ASV_23	50	14.29	0.02
*Lachnospira* ASV_488	21.43	—	0.02
*Peptoniphilus* ASV_54	50	11.43	0.01
*Peptoniphilus* sp. KHD5 ASV_120	28.57	2.86	0.02
*Peptostreptococcus* ASV_100	28.57	—	—
*Prevotella* 6 ASV_48	42.86	11.43	0.02
Prevotella bivia ASV_42	28.57	2.86	0.02
Prevotella disiens JCM 6334^*d*^	28.57	2.86	0.02
*Ruminococcaceae* UCG-002 ASV_161	21.43	—	0.02
*Ruminococcaceae* UCG-005 ASV_323	21.43	—	0.02
*Ruminococcus* 1 ASV_413	21.43	—	0.02
*Ruminococcus* 2 ASV_66	35.71	5.71	0.02

a ASVs in bold overlap with those identified in the best classification model (see Table 4).

b <32 weeks: stillbirth *n* = 15, live birth *n* =16; 32–36 weeks: stillbirth *n* = 10, live birth *n* = 15; >37 weeks: stillbirth *n* = 14, live birth *n* = 35. —, not detected.

c Fisher’s exact test (two-sided) was used.

d ATCC 29426 ASV_61.

10.1128/mbio.02036-22.3 FIG S3 Microbiota-based model to predict stillbirth (case) and live birth (control) in <32 weeks gestational age. Classification model based on Random Forest algorithm by using the abundance of the 3 ASVs. (a) Number of variables and mean squared error of the corresponding model. (b) The Random Forest model assigns a mean error rate or feature-importance score to each feature; this value indicates the extent to which each predictor contributes to the accuracy of the model. This model achieves an accuracy of 0.74. Download FIG S3, PDF file, 0.04 MB. Copyright © 2022 Vander Haar et al. 2022 Vander Haar et al. https://creativecommons.org/licenses/by/4.0/ This content is distributed under the terms of the Creative Commons Attribution 4.0 International license.

10.1128/mbio.02036-22.4 FIG S4 Microbiota-based model to predict stillbirth (case) and live birth (control) in ≥37 weeks gestational age. Classification model based on Random Forest algorithm by using the abundance of the 24 ASVs. (a) Number of variables and mean squared error of the corresponding model. (b) The Random Forest model assigns a mean error rate or feature-importance score to each feature; this value indicates the extent to which each predictor contributes to the accuracy of the model. This model achieves an accuracy of 0.78. Download FIG S4, PDF file, 0.04 MB. Copyright © 2022 Vander Haar et al. 2022 Vander Haar et al. https://creativecommons.org/licenses/by/4.0/ This content is distributed under the terms of the Creative Commons Attribution 4.0 International license.

Within stillbirth and live birth groups, respectively, significant differences were detected by gestational age as well (Tables 6 and 7). In the live birth group, a total of 13 ASVs detected in <32 weeks were either reduced or absent in >37 weeks. Four of these 13 ASVs belonged to *Fusobacterium*, with Fusobacterium nucleatum subsp. *animalis* ATCC 51191 identified as the best hit for three of them based on NCBI BLASTN. This is consistent with a previous report that subsp. *animalis* was the most prevalent F. nucleatum detected in intrauterine infection (17). In addition to F. nucleatum, several common oral anaerobes were also found to be specifically enriched in live early preterm births, including *Actinomyces*, Campylobacter, *Fusobacterium*, *Peptostreptococcus*, *Porphyromonas*, and *Prevotella*, suggesting a possible oral origin of infection (Table 7).

**TABLE 6:** ASVs in umbilical cord blood differentially detected between different gestational age groups within stillbirth groups

Stillbirth (cases)^*a*^			
ASV	<32 wks (%)	>37 wks (%)	*P* value^*b*^
*Blautia* ASV_163	—	28.57	0.04
*Blautia* ASV_178	—	50	0
*Dorea* ASV_118	—	35.71	0.02
*Peptostreptococcus* ASV_100	—	28.57	0.04
*Ruminococcus* 2 ASV_66	—	35.71	0.02
	**32–36 wks (%)**	**>37 wks (%)**	***P* value^*b*^**
*Bacteroides* ASV_29	—	50	0.02
*Blautia* ASV_178	—	50	0.02
*Faecalibacterium* ASV_121	—	42.86	0.02

a <32 weeks *n* = 15, 32–36 weeks *n* = 10, >37 weeks *n* = 14. —, not detected.

b Fisher’s exact test (two-sided) was used.

**TABLE 7:** ASVs in umbilical cord blood differentially detected between different gestational age groups within live births

Live births^*a*^			
ASV	<32 wks (%)	>37 wks (%)	*P* value^*b*^
*Actinomyces* ASV_239	18.75	—	0.03
Campylobacter ureolyticus ASV_79	37.5	2.86	0
*Corynebacterium* 1 ASV_201	18.75	—	0.03
*Ezakiella massiliensis* ASV_222	25	—	0.01
*Fenollaria timonensis* ASV_189	18.75	—	0.03
*Fusobacterium* ASV_159	18.75	—	0.03
*Fusobacterium* ASV_27	18.75	—	0.03
*Fusobacterium* ASV_5	31.25	—	0
*Fusobacterium* ASV_72	25	—	0.01
*Peptostreptococcu*s ASV_100	18.75	—	0.03
*Porphyromonas* ASV_110	25	2.86	0.03
Prevotella bivia ASV_34	25	—	0.01
Prevotella bivia ASV_42	25	2.86	0.03

a <32 weeks *n* = 16, >37 weeks *n* = 35. —, not detected.

b Fisher’s exact test (two-sided) was used.

## DISCUSSION

In this study, we demonstrate that the presence, abundance, and composition of bacterial DNA in cord blood are associated with stillbirth and vary by gestational age. Prediction models were generated to differentiate stillbirth and live birth. We identified organisms not previously associated with stillbirth and suggests that cord blood microbial signatures may be markers of adverse outcomes. These findings may lead to new ways for risk evaluation and identification of targets for prevention.

Our study has several strengths, including the use of data from a large, prospective, population-based, multisite case-control study. The study collected multiple biospecimens, and both the collection processes and the analyses of these specimens were rigorous, reliable, and well documented (18, 19). Researchers were blinded to the primary outcome throughout the course of specimen processing and sequencing, eliminating measurement bias. Placental pathology was performed in the majority of cases and controls. Furthermore, we utilized two complementing culture-independent approaches to analyze the cord blood microbiome.

Analysis of cord blood microbiome is challenging due to the low biomass, which is prone to both false positive (due to contamination) and false negative (loss of true signals) results. As contamination is a serious concern in analyzing utero samples (20), we conducted endpoint PCR of the full-length 16S rRNA, which is a less sensitive approach, thus reducing the likelihood of detecting low-level contaminations. The flipside is that, also due to its limited sensitivity, this approach may generate false negative results. Next-generation sequencing complements this weakness with significantly increased sensitivity, which in turn may produce false positive results. We included 12 blank controls for detecting and removing possible contamination during DNA extraction, library preparation, and sequencing in our data set. As a retrospective study, we were unable to control for contamination that may have occurred during sample collection. Nevertheless, the observation that the PCR positive samples had significantly higher total read counts provides corroboration between these two methods, strengthening the veracity of our conclusion. The use of ASVs, instead of OTU (operational taxonomic units), provided improved resolution, down to the level of single-nucleotide differences (21), thus allowing discovery and tracking of potential pathogens. These approaches ensure identification of true signals associated with potential infections.

The current findings indicate that the bacterial infection rate in stillbirth may be severely underestimated in previous studies. Both the bacterial DNA prevalence and abundance were significantly increased in stillbirth than in live birth, indicating enhanced severity of infection in stillbirth. Among them, GBS was the major contributor. As GBS colonizes the maternal genital track, it likely invades into the intrauterine cavity via an ascending mechanism, although hematogenous infection cannot be ruled out. Although previous meta-analysis identified GBS as an important risk predictor of stillbirth, it was only attributed to 1% of stillbirths worldwide and 4% in Africa (19). Our results indicate that these numbers may be severely underestimated. GBS has been previously cultivated from neonatal blood of preterm infants, further confirming the validity of our findings in fetal blood (10).

Unique microbiome signatures were detected in stillbirth, as well as in live early preterm birth. The latter appeared to be dominated by oral anaerobes, suggesting a possible oral route of transmission. This is in support of previous finding of similarities between placental and oral microbiomes (22). F. nucleatum was detected as the most prevalent species in early preterm birth, consistent with the report that intrauterine infection of F. nucleatum is inversely related to gestational age (23). F. nucleatum has been implicated in a spectrum of adverse pregnancy outcomes, including stillbirth, preterm birth, as well as neonatal sepsis (7, 8, 10). Animal studies showed that oral F. nucleatum is capable of translocating hematogenously to the pregnant uterus, inducing TLR4-mediated maternal inflammatory responses leading to fetal demise (24–26). This process requires a unique virulence factor, FadA, which exhibits amyloid-like properties under stress and disease conditions (27). Amyloid FadA serves as a molecular switch to convert the microorganism from a benign commensal to virulent pathogen (28). Detection of common oral anaerobes in early preterm birth suggests that oral-hematogenous translocation may play a significant role.

One limitation of the study is that only a subset of stillbirths had umbilical cord blood available, which are biased toward more recent deaths. The possibility exists that the increased microbial abundance and diversity may be the result rather than the cause of stillbirth. Microbial detection in cord blood in term live births may suggest the existence of a resident microbiome, similar as in other utero samples (20). Further study is required to confirm this finding.

In conclusion, our findings underscore the utility of complementing methods for detecting possible infections where low-biomass organisms may be present. The strong association we uncover between ASV-level microbial signatures in umbilical cord blood with stillbirth and early preterm birth lends credence to this approach. Analysis of cord blood will identify microbes capable of invading into deeper fetal-placental compartments and potentially improve our ability to prevent stillbirth and early preterm birth. Future study will be warranted to evaluate the potential source of infection, and in particular, the implication of oral-hematogenous translocation in early pregnancy complications. In addition, it was reported previously that placental infection and inflammation in mice could be suppressed by dietary supplementation of omega-3 fatty acids (26). It will be worth testing if omega-3 supplementation can reduce stillbirth and early preterm birth in humans.

## MATERIALS AND METHODS

### Study design.

**FIG 5:**
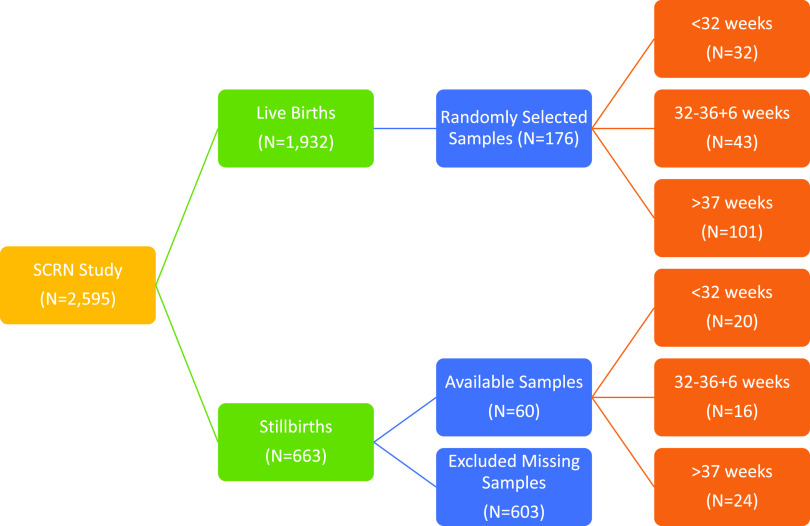
Flowchart showing breakdown of study population and number of samples in each category by gestational age.

This cross-sectional case-control study utilized umbilical cord blood specimens from the biospecimen repository of the Stillbirth Collaborative Research Network (SCRN), conducted from March 2006 to September 2008, with follow-up through December 2009, at 59 hospitals associated with five clinical sites (29). The cases were defined as stillbirths, and controls as liveborn infants. We included in our analyses all stillbirths (N = 60) and preterm live births (N = 75) from the original study for which umbilical cord blood specimens were available, as well as randomly selected live term births (gestational age >37 weeks; *n* = 101) (Fig. 5). The research protocol was approved by the institutional review board (IRB) at Columbia University Irving Medical Center. De-identified demographic data linked to the samples was obtained through the National Institute of Child Health and Development Data and Specimen Hub (DASH) following IRB approval. Laboratory data were collected and managed using Research Electronic Data Capture (REDCap) tools hosted at Columbia University (30).

### Bacterial DNA extraction and detection.

Samples were processed in random order and without knowledge of clinical outcomes. An aliquot of 0.5 mL of de-identified cord blood specimens were spun at 13,000 *g* for 15 min. The pellets were used for DNA extraction using the QIAamp BiOstic Bacteremia DNA Kit (Qiagen). The full-length 16S rRNA gene was amplified by PCR using universal primers A17F (GTTTGATCCTGGCTCAG) and 1512R (TACCTTGTTACGACTT), which can detect the majority of eubacteria, as previously described (31). Negative controls using sterile PBS were included with each set of DNA extraction and PCR to control for contamination.

### 16S rRNA gene analysis.

Hypervariable region V4 of the 16S rRNA gene was amplified using the 515F and 806R primers (32) and sequenced using the Illumina MiSeq platform with 2 × 250 mode at the Alkek Center for Metagenomics and Microbiome Research (CMMR) at Baylor College of Medicine. Amplicon sequence variants (ASVs) were obtained by denoising using the DADA2 (11) denoise-paired command in QIIME 2 (33). Spurious ASVs were further removed by abundance filtering (12). All ASVs were compared with human genome and nr databases to remove sequences from nonprokaryotic sources. Buffer contaminants were identified in the blank controls using the decontam R package (14) and removed from all samples. The data were then rarified to 1,000 reads/sample for analyses. Samples with fewer reads than the rarefication depth were removed. Principal coordinates analysis (PCoA) was performed by the R package “ape” (34). Random Forest analysis was performed and cross-validated using the R “RandomForest” package (35) and the “rfcv” function, respectively, to build the classification model. A phylogenetic tree of ASVs was built using the QIIME 2 commands alignment mafft, alignment mask, phylogeny fastree, and phylogeny midpoint-root. Taxonomy assignment of ASVs was performed by the q2-feature-classifier plugin in QIIME 2 based on the SILVA database (release 132) (36).

### Statistical analyses.

Differences in baseline characteristics between cases and controls were analyzed by the chi-squared test or Fisher’s exact test, as appropriate, for categorical variables, and the Student's *t* test for continuous variables. Non-Gaussian data were analyzed using the Wilcoxon rank-sum test. Univariate logistic regression analysis was used to determine significant predictors of the birth outcomes of interest. A multivariable logistic regression model was utilized to evaluate the independent effect of those predictors while adjusting for demographic factors. In addition, as the total read count is highly skewed to the right, a log-transformation was used in all regression analyses. Models incorporating histologic chorioamnionitis did not include multiple gestations, as histopathologic data were not available for these samples. All analyses were performed in SAS Version 9.4 (SAS Institute, Inc., Cary, NC) and R Version 3.6.0 (R Foundation for Statistical Computing, Vienna, Austria). Alpha- and beta-diversity metrics were calculated using QIIME2 (33), with significance of differences assessed by the Mann-Whitney U test and PERMANOVA test, respectively. Fisher’s exact test (two-sided) and Mann-Whitney test (two-sided) were used to compare the prevalence and abundance of the ASVs between different groups.

### Data availability.

The raw sequencing data of 16S rRNA the gene V4 region has been deposited to the sequence read archive at NCBI under the BioProject ID PRJNA778571.

Scripts and command lines related to the current study are freely available from the corresponding author (Y.W.H.) upon request.
