# Supplementary figures and images for: Microbial Analysis of Umbilical Cord Blood Reveals Novel Pathogens Associated with Stillbirth and Early Preterm Birth
Source: mBio. 2022 Aug 22;13(5):e02036-22. doi: 10.1128/mbio.02036-22 (PMC9600380; doi:10.1128/mbio.02036-22)

Figure S1

**a**

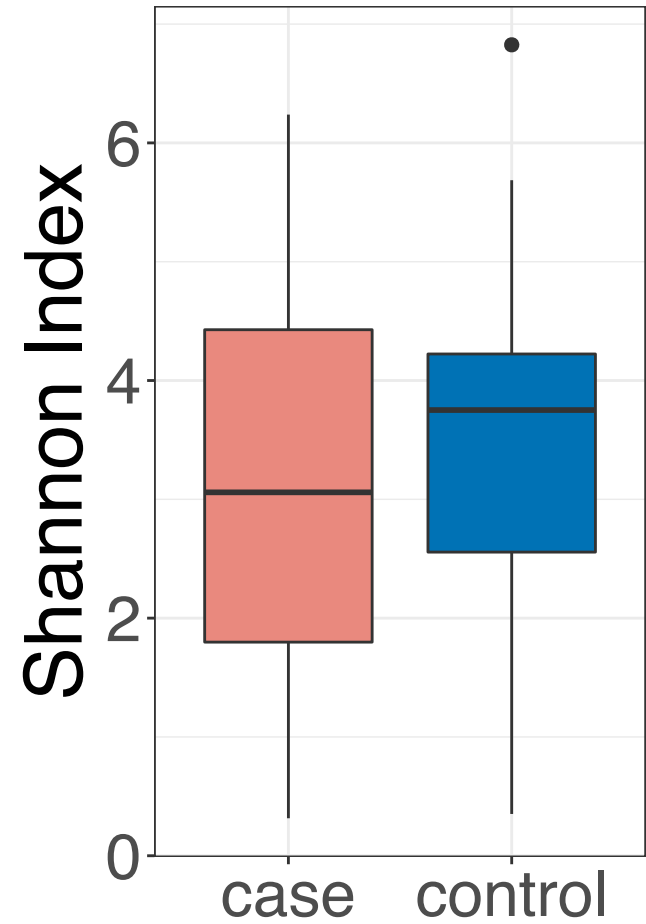

**b**

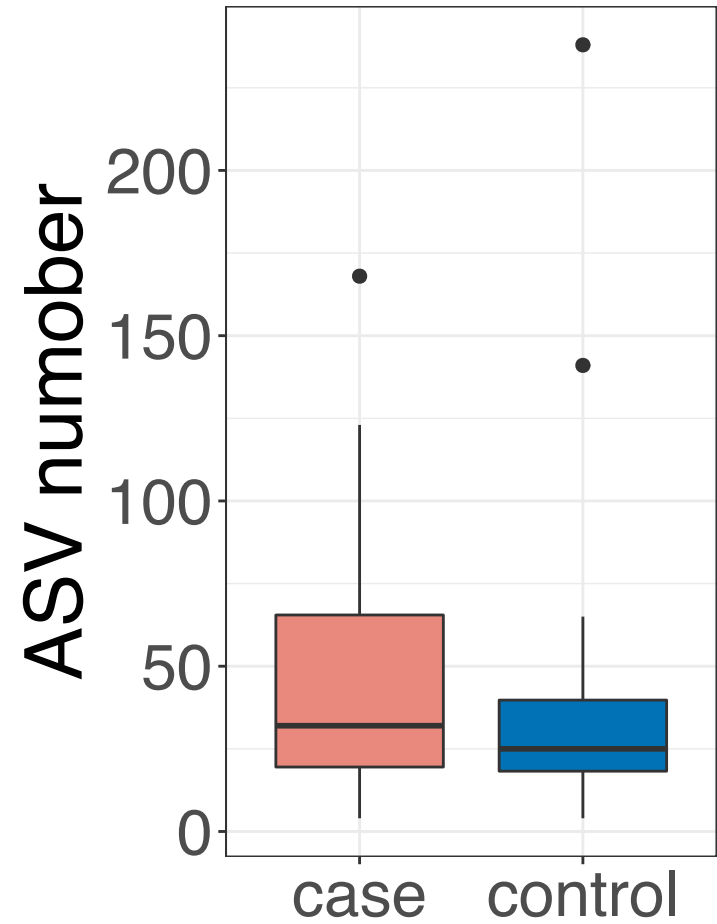

Supplement: FIG S1 [file mbio.02036-22-s0001.pdf]

Figure S2

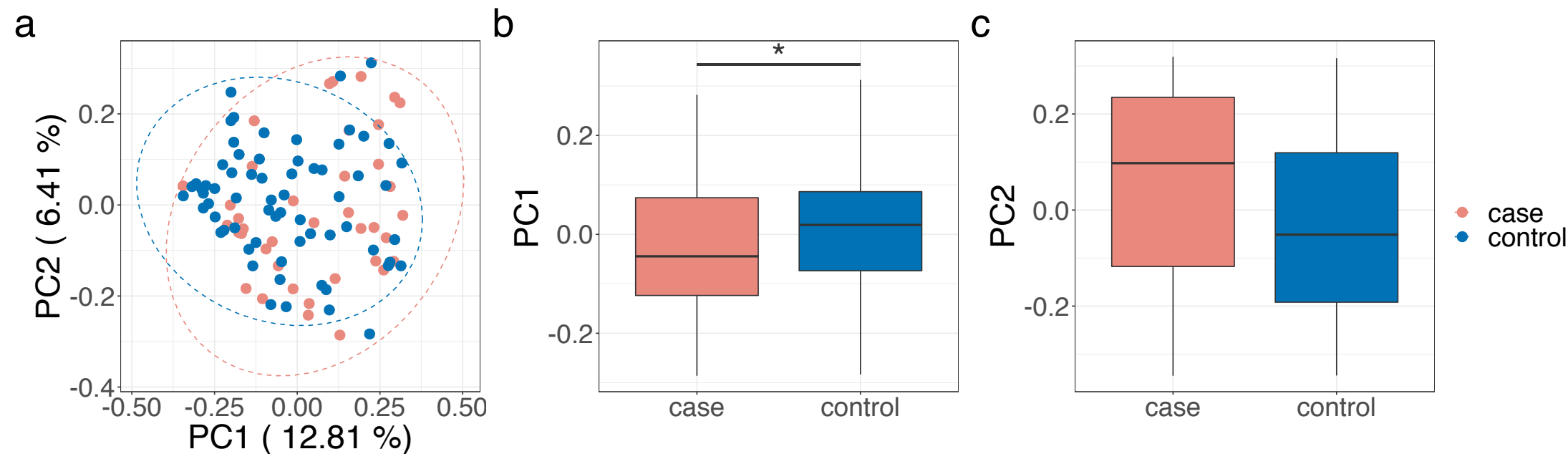

Supplement: FIG S2 [file mbio.02036-22-s0002.pdf]

Figure S3.

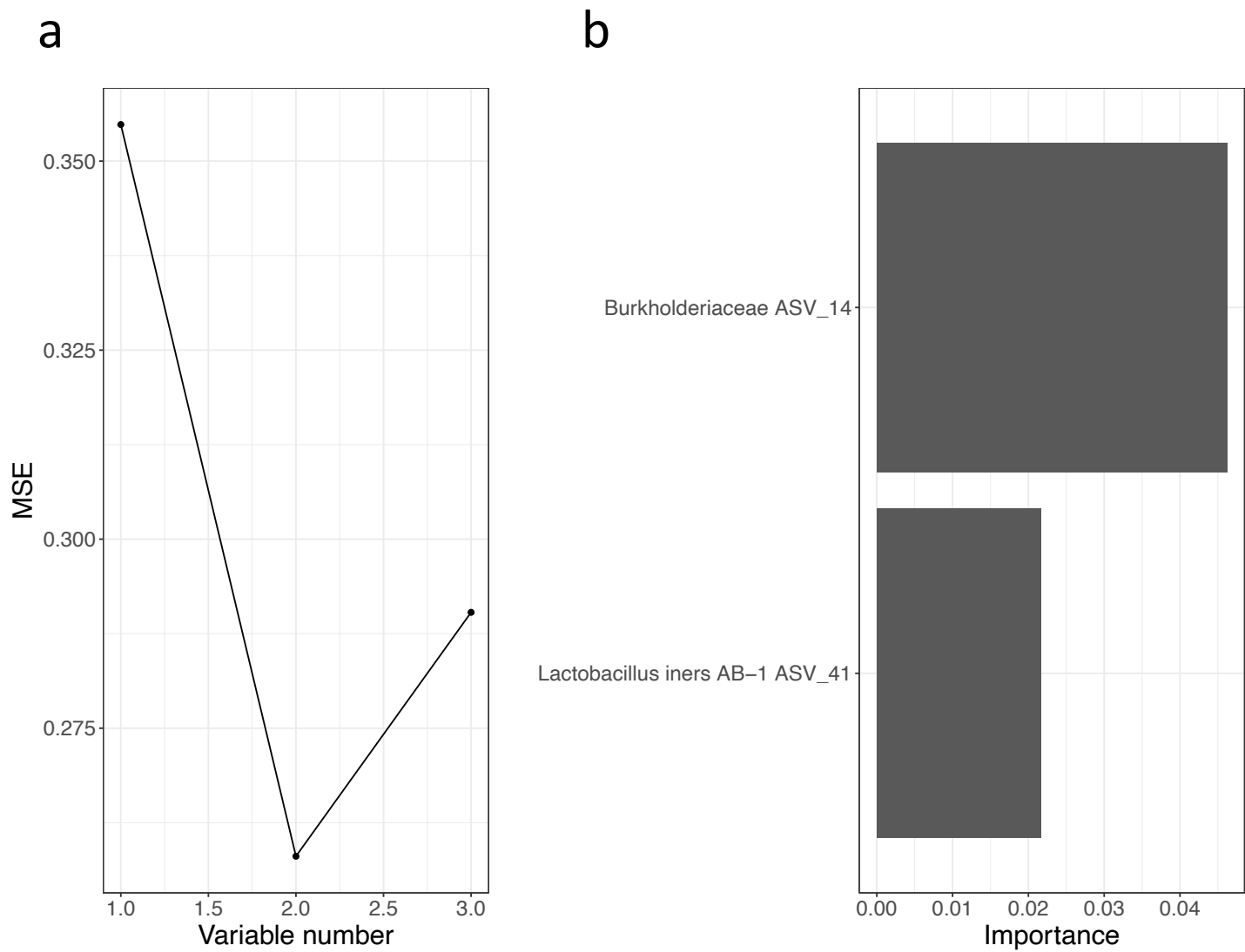

Supplement: FIG S3 [file mbio.02036-22-s0003.pdf]

Figure S4.

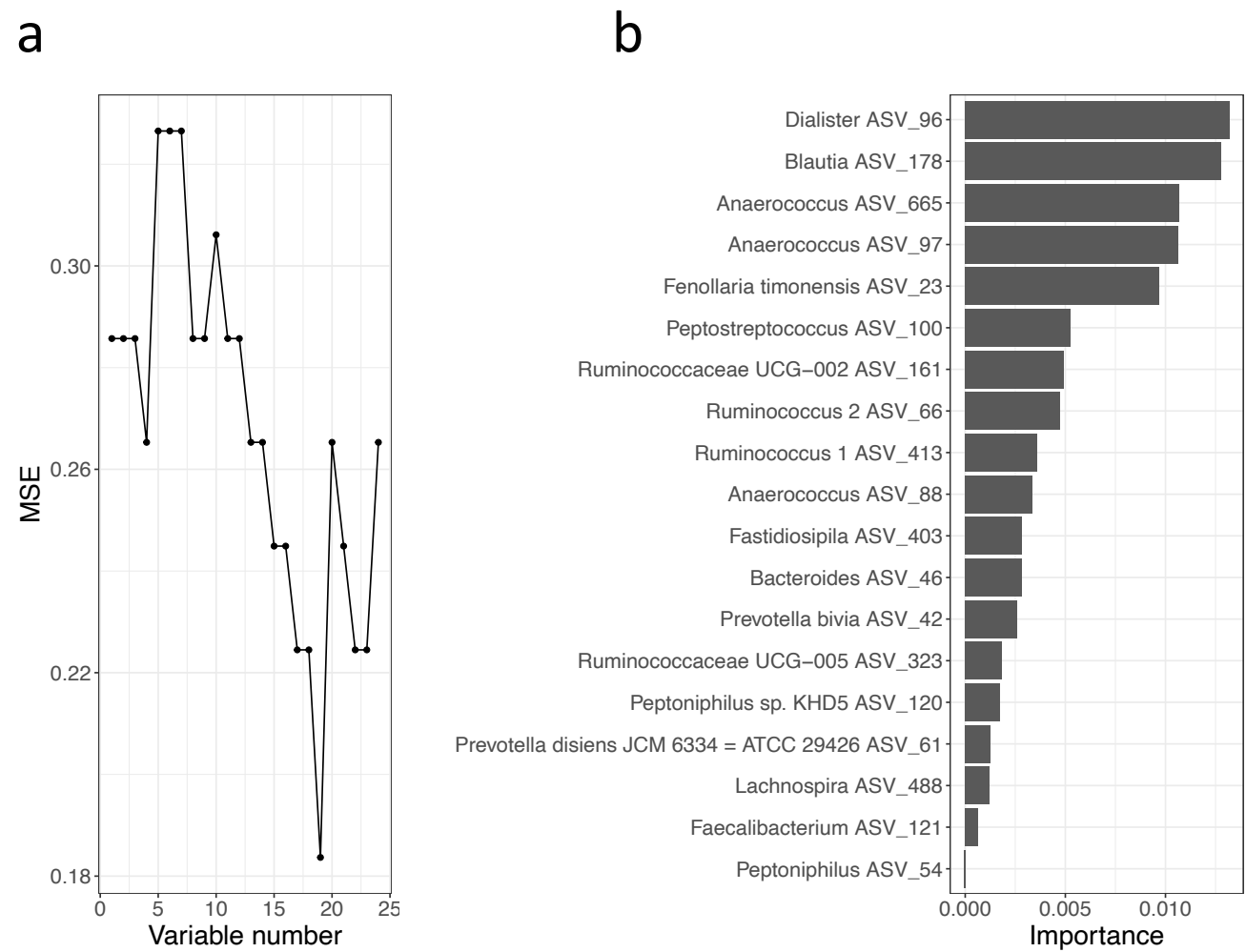

Supplement: FIG S4 [file mbio.02036-22-s0004.pdf]
